# Behavioural and objective vestibular assessment in persons with osteoporosis and osteopenia: a preliminary investigation^☆^

**DOI:** 10.1016/j.bjorl.2017.08.013

**Published:** 2017-09-21

**Authors:** Aditi Gargeshwari, Raghav Hira Jha, Niraj Kumar Singh, Prawin Kumar

**Affiliations:** All India Institute of Speech and Hearing, Department of Audiology, Manasagangothri, Karnataka, India

**Keywords:** Osteoporosis, Osteopenia, Bone mineral density, cVEMP, oVEMP, Balance assessment, Osteoporose, Osteopenia, Densidade mineral óssea, cVEMP, oVEMP, Avaliação de equilíbrio

## Abstract

**Introduction:**

Calcium is vital for the functioning of the inner ear hair cells as well as for the neurotransmitter release that triggers the generation of a nerve impulse. A reduction in calcium level could therefore impair the peripheric vestibular functioning. However, the outcome of balance assessment has rarely been explored in cases with osteopenia and osteoporosis, the medical conditions associated with reduction in calcium levels.

**Objective:**

The present study aimed to investigate the impact of osteopenia and osteoporosis on the outcomes of behavioural and objective vestibular assessment tests.

**Methods:**

The study included 12 individuals each in the healthy control group and osteopenia group, and 11 individuals were included in the osteoporosis group. The groups were divided based on the findings of bone mineral density. All the participants underwent behavioural tests (Fukuda stepping, tandem gait and subjective visual vertical) and objective assessment using cervical and ocular vestibular evoked myogenic potentials.

**Results:**

A significantly higher proportion of the individuals in the two clinical groups’ demonstrated abnormal results on the behavioural balance assessment tests (*p* < 0.05) than the control group. However, there was no significant difference in latencies or amplitude of cervical vestibular evoked myogenic potential and oVEMP between the groups. The proportion of individuals with absence of ocular vestibular evoked myogenic potential was significantly higher in the osteoporosis group than the other two groups (*p* < 0.05).

**Conclusion:**

The findings of the present study confirm the presence of balance-related deficits in individuals with osteopenia and osteoporosis. Hence the clinical evaluations should include balance assessment as a mandatory aspect of the overall audiological assessment of individuals with osteopenia and osteoporosis.

## Resumo

**Introdução:**

O cálcio é vital para o funcionamento das células ciliadas, assim como para a liberação dos neurotransmissores que desencadeiam um impulso nervoso. Uma redução nos níveis de cálcio poderia, portanto, prejudicar o funcionamento vestibular periférico. No entanto, a avaliação do equilíbrio tem sido raramente explorada em casos de osteopenia e osteoporose, condições médicas associadas à redução dos níveis de cálcio.

**Objetivo:**

O presente estudo teve como objetivo investigar o impacto da osteopenia e da osteoporose nos resultados dos testes de avaliação comportamental e vestibular objetiva.

**Método:**

O estudo incluiu 12 indivíduos nos grupos controle e grupo de osteopenia e 11 indivíduos no grupo da osteoporose. Os grupos foram divididos com base nos achados da densidade mineral óssea. Todos os participantes foram submetidos a testes comportamentais (Prova dos Passos de Fukuda, Marcha em tandem e Vertical Visual Subjetiva) e à avaliação objetiva com o uso de potenciais evocados miogênicos vestibulares cervical e ocular (cVEMP e oVEMP).

**Resultados:**

Uma proporção significativamente maior de indivíduos nos dois grupos com condições clínicas mostrou resultados anormais nos testes de avaliação comportamental e do equilíbrio (*p* < 0,05) do que o grupo controle. Embora não tenha havido diferença significativa nas latências ou na amplitude de cVEMP e oVEMP entre os grupos, a proporção de indivíduos com ausência de oVEMP foi significativamente maior no grupo da osteoporose do que nos outros dois grupos (*p* < 0,05).

**Conclusão:**

Os resultados do presente estudo demonstram a presença de déficits de equilíbrio em indivíduos com osteopenia e osteoporose. Assim, as avaliações clínicas gerais e audiológicas de indivíduos com osteopenia e osteoporose deveriam incluir a avaliação do equilíbrio como um aspecto obrigatório.

## Introduction

Osteoporosis is a medical condition in which the bones become brittle and fragile due to loss of tissue, typically as a result of hormonal changes, deficiency of calcium and/or deficiency of vitamin D.^1^, ^2^ Osteopenia is also a medical condition in which the protein and mineral content of bone tissue is reduced, but less severely than in osteoporosis.^1^, ^2^ These two pathologies thus represent a continuum along the bone mineral density measure.

Loss of bone mineral is a phenomenon that takes place in all human beings. This daily removal of small amounts of bone mineral, a process called resorption, must be balanced by an equal deposition of new mineral if bone strength is to be preserved.^3^ When this balance tips towards excessive resorption, bones weaken and can become brittle and prone to fracture over time. This continual resorption and redeposition of bone mineral, often termed bone remodelling, is intimately tied to the pathophysiology of osteoporosis.^3^

The balance between bone resorption and bone deposition is determined by the activities of two principle cell types, osteoclasts and osteoblasts.^4^ The alterations in the intracellular calcium ion (Ca^2+^) concentrations regulate differentiation and functions of osteoclasts.^4^ Further, the changes in intracellular Ca^2+^ concentrations are known to function as universal triggers of diverse signalling pathways, including enzyme activation, cell survival and cell differentiation.^4^ This would imply that a reduction in the Ca^2+^ concentration is not only likely to affect cell differentiation and survival but also affect the functioning of various neural pathways. This combination increases the brittleness of various bones of the body.

According to the International Osteoporosis Foundation (IOF), 40% of women in the world have fractures due to osteoporosis during their lifetime.^3^ Fractures are associated with increased morbidity/mortality and diminished quality of life in a variety of ways, including decline in physical and emotional functioning.^5^ Falls are responsible for 90% of hip fractures^6^ and are the sixth leading cause of death among patients aged 65 years and older.^7^ In addition to the high mortality, there are other deleterious consequences of falls, including restriction of mobility, disability, social isolation, insecurity and fear, often inducing a cascade of events that are harmful to health and quality of life in the elderly.^8^, ^9^ Research suggests that altered balance is the greatest contributor towards falls in the elderly, with a high correlation between balance deficit and incidence of falls.^10^ Furthermore, the programs targeting mainly balance training have been demonstrated to be effective in prevention of falls among the elderly,^11^ many of whom might be affected with low bone mineral density.

It is well documented that calcium is vital for functioning of various different organ systems in our body, including the hearing and vestibular systems.^12^, ^13^ Further, Madureira et al. observed that the older adults who experienced falls had impaired balance function.^14^ Since old age is found to be associated with reduction in Ca^2+^ concentration, there is a likelihood that the impairment of balance function in these individuals might have been caused by the decreased calcium concentration (osteopenia/osteoporosis).^3^, ^4^ Alternatively, there are theories suggesting that reduced vestibular functioning could actually be the cause of bone remodelling and thereby osteoporosis, the proof for which originates from the experiments in lower animals like rats and mice.^15^, ^16^ Therefore it appears that there could be a relationship between the reduced levels of calcium and balance sustenance ability. However, this aspect has been minimally investigated.

Altered bone composition results in structural changes of the bones such as kyphosis,^17^ which can also lead to balance issues. In a study by Abreu et al. it was found that women with osteoporosis had worse balance and maximum oscillation in evaluation of balance using the Polhemus system in four upright postural situations when compared to non-osteoporotic women.^18^ In the group with osteoporosis, Lynn et al. observed larger sway amplitudes and inappropriate use of balance maintenance strategies than the controls when evaluated using the Computerised Dynamic Posturography (CDP).^19^ Recent evidence suggests that the density of otoconia is reduced in adult female osteoporotic rats.^20^ Since otoconia are present in the utricle and saccule, which are vital for generating the Vestibula revoked Myogenic Potentials (VEMP),^21^, ^22^ impairment of VEMP could be expected in these patients. However, there are no reports to validate this observation. Although there is sporadic evidence to suggest the presence of vestibular deficits in individuals with osteoporosis, there is a scarcity of studies to suggest whether osteopenia is associated with similar deficits as osteoporosis. This therefore highlights the need to study the vestibular system functioning in individuals with osteopenia and osteoporosis. Therefore, the present study aimed to investigate the impact of osteopenia and osteoporosis on the outcomes of behavioural and objective vestibular assessment tests.

## Methods

### Participants

All procedures performed in studies involving human participants were strictly in accordance with the ethical standards of the institutional research committee. The present study included 35 participants who had undergone bone mineral density test. The WHO (2004) has recommended a diagnosis of osteopenia when the *T*-score (outcome of bone mineral density test) is between −1.1 to −2.5 and osteoporosis when the *T*-score is ≥−2.6 (in the negativity), respectively.^23^ Those with *T*-scores better than −1.1 (towards positivity) are considered normal. A total of 37 participants were included in the study; however 2 females, both with osteoporosis, could not maintain eye elevation necessary for oVEMP, as they found the task too straining, blinked often, and therefore they were excluded. Finally, after exclusions and based on the *T*-scores the study had 12 individuals each in the healthy control group (6 males and 6 females; age range: 42–75 years, mean age = 58 years, SD = 10.5) and osteopenia group (6 males and 6 females; age range: 45–76 years, mean age = 64.4, SD = 9.6) whereas 11 individuals were there in the osteoporosis group (5 males and 6 females; age range: 47–76 years, mean age = 62.1, SD = 8.9). There was no significant difference in age between the groups (*p* > 0.01, MANOVA). Since the objective vestibular assessment and conductive pathologies are known to cause absence of cVEMP and oVEMP, persons with conductive pathologies were excluded. Further, individuals with neurological deficits were excluded after consultation with a neurologist. The screening by an experienced otolaryngologist helped to exclude persons with other known causes of vestibular pathologies. Persons with a history of occupational noise exposure and exposure to ototoxic medication were also excluded from the study. All participants in the study had normal levels of thyroid even though all the female participants in the study were post-menopausal. None of the participants had undergone any treatment for altered bone remodelling, as they were recently diagnosed. The male participants of the study had an urban sedentary life style, however 5 female participants in each group were housewives. None of the participants were chronic smokers or alcoholics. All participants signed the informed written consent form before being recruited to the study and they were not paid for their participation in the study. Informed consent was obtained from all individual participants included.

### Instrumentation and environment

In the present study, all the behavioural tests were carried out in a well-illuminated and air-conditioned room. These included Fukuda Stepping Test (FST), Tandem Gait Test (TGT) and Subjective Visual Vertical (SVV) test. The objective tests were carried out in a sound-treated room with noise levels well within the permissible limits recommended by American National Standards Institute (ANSI S3.1, 1991).^24^ The objective tests included cervical and ocular VEMP (cVEMP and oVEMP). Cervical and Ocular VEMPs were recorded using Biologic Navigator Pro version 7.2.1 with SINSER-012 insert earphones.

### Procedure

For the FST, the participants were instructed to stretch both their arms in front of them and march with their eyes closed at the same place for 50 steps at a rate of 1 step. The angle of deviation >45° in either direction and/or distance of deviation >1 m was considered abnormal as reported previously.^25^ For the TGT, the participants were asked to imagine a straight line on the floor and walk heel-to-toe along it for 30 s. Loss of balance or raising of arms to ensure balance sustenance before 30 s was deemed abnormal, as reported previously.^26^ During the SVV, participants were handed a bucket with a bright strip placed inside it. They were instructed to place their head as inside the bucket as comfortable and align the position of the strip in their perceived vertical position. The bucket was handed at different angles to the participants in order to reduce bias. The angle of deviation from the actual vertical was noted by a pointer indicating the angles on a 360° protractor attached to the back of the bucket. A >3° deviation of the perceived vertical from the true vertical was deemed to be abnormal result. This is as per the previously reported findings.^27^, ^28^

Before recording the evoked potentials, the electrode placement sites were scrubbed using a commercially available abrasive gel. The disc type surface electrodes were placed using commercially available conduction paste and secured in place using surgical plaster. The absolute and inter-electrode impedance was maintained below 5 kΩ and 2 kΩ respectively for both cVEMP and oVEMP.

For the cVEMP recordings, the electrodes were placed at the sternoclavicular junction (inverting), superior 1/3rd of sternocleidomastoid muscle (non-inverting) and forehead (ground). For the activation of the sternocleidomastoid muscle, the participants were asked to turn their head away from the ear of stimulation. The recording was done for tone bursts of 500 Hz, 750 Hz and 1000 Hz with 1 ms rise/fall times and a 2 ms plateau time. The tone-bursts were presented to the ear at an intensity of 125 dB peSPL using a stimulation rate of 5.1 Hz. A 1–1500 Hz band-pass filter was used and the responses were averaged for 200 sweeps. The pre-stimulus rectification was used to control for the effects of variations in the electromyographic activity between the cVEMP recordings.

The oVEMP was obtained by placing the non-inverting electrode on the skin surface 1 cm below the centre of the lower eyelid, inverting electrode 2 cm below the non-inverting and ground on the forehead, as found appropriate and used previously.^29^, ^30^ The participants were instructed to maintain a 30° upward centre gaze by staring at a strip placed at the appropriate elevation. The recording was done using tone bursts of 500 Hz, 750 Hz and 1000 Hz with 1 ms rise/fall times and a 2 ms plateau time. The tone bursts were delivered to the ear canal at an intensity of 125 dB peSPL using a repetition rate of 5.1 Hz. A 1–1000 Hz band-pass filter was used and the responses were averaged for 200 sweeps.

In order to compare the results of vestibular evaluation against the better known audiological results in persons with reduced bone-mineral density, audiological evaluations were carried out. These included pure-tone air- and bone-conduction audiometry, Speech Reception Threshold (SRT), Speech Identification Scores (SIS), immittance evaluation (tympanogram type, acoustic reflex threshold and resonance frequency) and distortion product oto-acoustic emissions.

### Statistical analyses

The Equality of test for proportions was carried out to find the proportion of participants showing abnormal findings. This was also done to investigate the between groups difference in response rate (number of ears with presence of cVEMP/oVEMP at 500 Hz) and frequency tuning. Separate one-way repeated measure ANOVA for ears with group as the between subjects factor was done for latencies of individual peaks and peak-to-peak amplitude. Further, one-way repeated measures ANOVA was done for ears with group on the between subject factor for pure-tone average, speech recognition threshold, speech identification scores and static compliance. The Equality of test for proportions was done to find the differences if any in the response rates of acoustic reflex and DPOAEs. Chi-square analyses were done to investigate the association between the groups and the outcomes of various evaluations.

## Results

A detailed structured case history was obtained from all the participants in each of the three groups. It was found that only one participant in the control group reported vestibular symptoms (swaying while walking). None of the other participants in the control group reported of any vestibular symptom such as vertigo, swaying and imbalance. These symptoms were reported by 4 participants (3 vertigo and 1 imbalance) in the osteopenia group and 7 participants (5 vertigo and 2 imbalances) in the osteoporosis group. In order to investigate the statistical significance of the above mentioned observations from the case history, the Equality of test for proportions was done. A significantly higher proportion of individuals with osteoporosis were found to exhibit vestibular symptoms than the control group (*Z* = 2.78, *p* = 0.005). However, there was no significant difference in the proportion of individuals with vestibular symptoms between the osteopenia group and the control group (*Z* = 1.50, *p* = 0.15) and also between the osteopenia group and the osteoporosis group (*Z* = 1.45, *p* = 0.14).

### Comparison between the groups on behavioural tests

A deviation of the perceived vertical of >3° on either side of the actual vertical has been considered an abnormal result on SVV.^27^, ^28^ Considering this normative, the abnormal results were found on SVV in a significantly higher proportion of individuals with osteoporosis (*Z* = 2.40, *p* = 0.01) and osteopenia (*Z* = 1.88, *p* = 0.05) than the control group. However, there was no significant difference between the osteopenia group and the osteoporosis group (*Z* = 0.61, *p* = 0.53).

The results on FST was considered abnormal when there was a deviation of >45° on either side and/or a distance of more than 1 m from the starting point.^25^ Considering this normative, significantly higher (marginally) proportion of individuals with osteoporosis had abnormal results than the healthy controls on FST, as revealed by the Equality of test for proportion (*Z* = 1.82, *p* = 0.06). The comparison between the other groups revealed no significant difference (*p* > 0.1).

**Figure 1:**
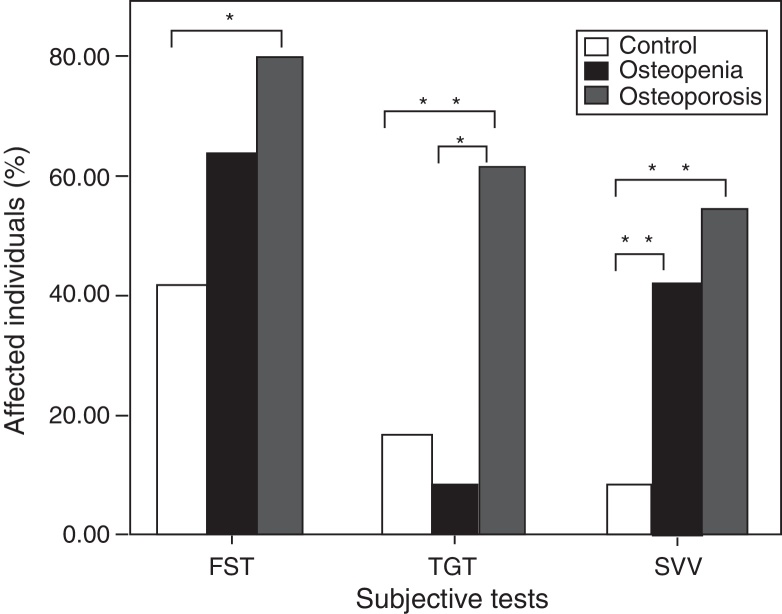
Percentage of participants with abnormal responses for each of the three subjective tests in each group. One star indicates marginally significant difference (0.05 > *p* < 0.1) between the groups and two stars indicate highly significant difference (*p* < 0.01) between the groups.

The inability to perform a balanced heel-to-toe walk (e.g., tendency to fall, raising hands to maintain balance or stumbling) was considered an abnormal result on TGT.^26^ The osteoporosis group had significantly higher proportions of abnormal results on TGT than the healthy controls (*Z* = 2.10, *p* = 0.03) or osteopenia group (*Z* = 2.59, *p* = 0.09). There was no significant difference between the other groups (*p* > 0.1). Fig. 1 shows the percentage of individuals with abnormal results in each group on the behavioural tests.

### Comparison between the groups on cVEMP

**Figure 2:**
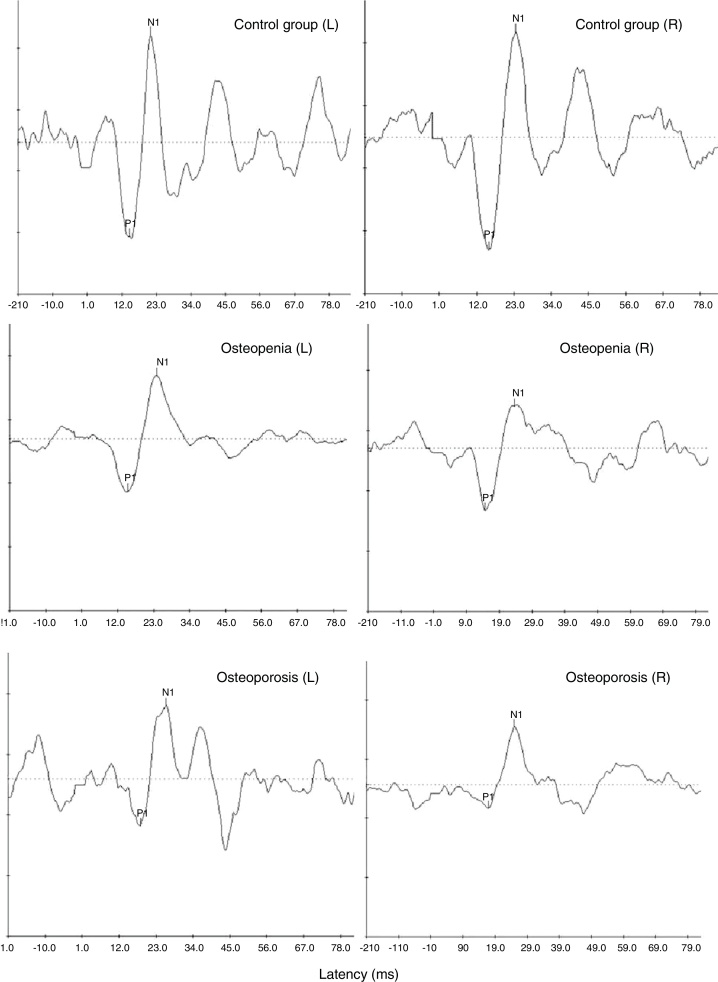
Representative cVEMP waveforms obtained from one participant with normal bone mineral density (top-most panel) and a participant each with osteopenia (middle panel) and osteoporosis (bottom-most panel).

Ipsilateral cVEMPs were recorded from both ears of all the participants in each of the groups. Fig. 2 shows the representative waveforms of cVEMP from one participant in each of the three groups. The mean, median and standard deviation of latencies and peak-to-peak amplitude of 500 Hz tone-burst evoked cVEMP were calculated and have been mentioned in Table 1.

**Table 1:** Mean, median and standard deviation of latencies of P1, N1 and peak-to-peak amplitude of 500 Hz tone-burst evoked cVEMP from the three groups of participants.

cVEMP	Control group (*n* = 21)			Osteopenia (*n* = 24)			Osteoporosis (*n* = 17)		
	$\bar{x}$	*M*	SD	$\bar{x}$	*M*	SD	$\bar{x}$	*M*	SD
P1	15.80	16.01	1.78	16.06	16.17	1.23	16.12	16.13	1.65
N1	23.81	23.77	2.95	24.26	24.23	1.90	23.69	22.92	2.48
P1N1	12.82	11.38	7.49	12.15	11.09	6.79	9.44	6.13	6.66

$\bar{x}$, mean; *M*, median; SD, standard deviation; P1, latency of P1 peak in milliseconds; N1, latency of N1 peak in milliseconds; P1N1, peak-to-peak amplitude of cVEMP in microvolts; cVEMP, cervical vestibular evoked myogenic potential.

The within and between groups comparison of various parameters of cVEMP was achieved using one-way repeated measures ANOVA for ears with group as the between subjects factor. Even though there was a trend for smaller amplitude in the two clinical groups than the control groups (smallest amplitude in osteoporosis), the results of one-way repeated measures ANOVA revealed no significant main effect for within and between groups comparison on any of the above mentioned parameters of cVEMP (*p* > 0.1).

### Comparison between the groups on oVEMP

**Figure 3:**
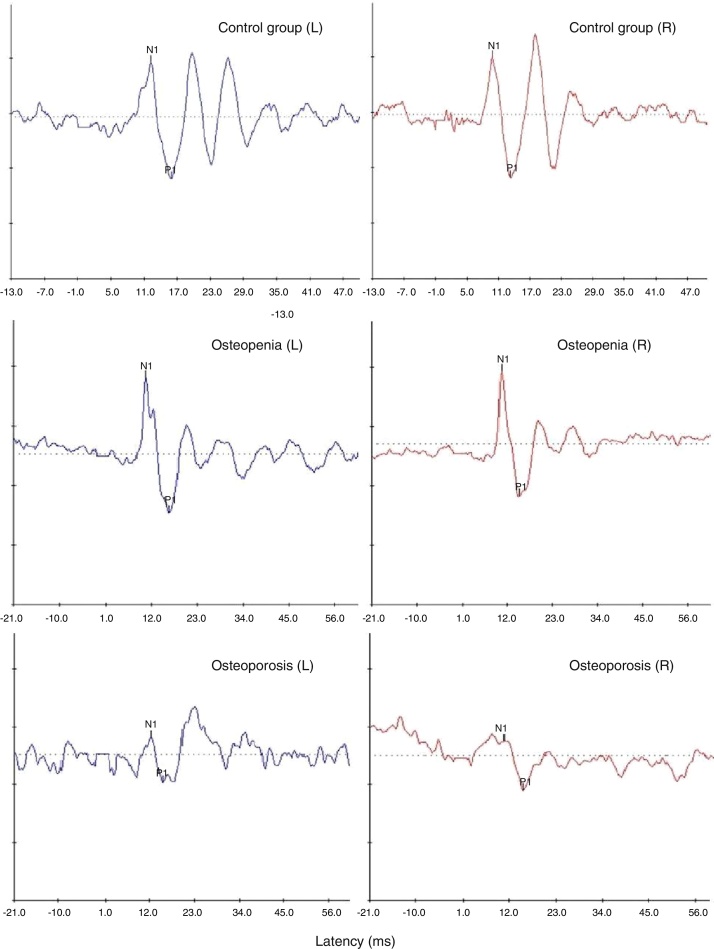
Representative oVEMP waveforms obtained from one participant with normal bone mineral density (top-most panel) and a participant each with osteopenia (middle panel) and osteoporosis (bottom-most panel).

Ipsilateral oVEMPs were recorded from both ears of all the participants in each of the groups. Fig. 3 shows the representative waveforms of oVEMP from one participant in each of the three groups. The mean, median and standard deviation of latencies and peak-to-peak amplitude of 500 Hz tone-burst evoked oVEMP were calculated and have been mentioned in Table 2.

**Table 2:** Mean, median and standard deviation of latencies of N1, P1 and peak-to-peak amplitude of 500 Hz tone-burst evoked oVEMP from the three groups of participants.

oVEMP	Control group (*n* = 21)			Osteopenia (*n* = 24)			Osteoporosis (*n* = 17)		
	$\bar{x}$	*M*	SD	$\bar{x}$	*M*	SD	$\bar{x}$	*M*	SD
N1	11.72	11.87	1.40	11.31	11.78	1.92	13.10	12.91	2.38
P1	16.89	16.77	1.94	17.09	17.12	2.81	17.75	17.92	1.03
N1P1	2.41	2.69	1.50	2.28	2.08	2.98	1.45	1.74	3.82

$\bar{x}$, mean; *M*, median; SD, standard deviation; N1, latency of N1 peak in milliseconds; P1, latency of P1 peak in milliseconds; N1P1, peak-to-peak amplitude of oVEMP in microvolts; oVEMP, ocular vestibular evoked myogenic potential.

There was a tendency for longer latencies and smaller peak-to-peak amplitudes in the two clinical groups (longest latencies and smallest amplitude in osteoporosis) than the control group. However, the results of one-way repeated measures ANOVA for ears with group as the between subjects factor revealed no significant main effect of ear and group on any of the above-mentioned parameters of oVEMP (*p* > 0.1).

**Figure 4:**
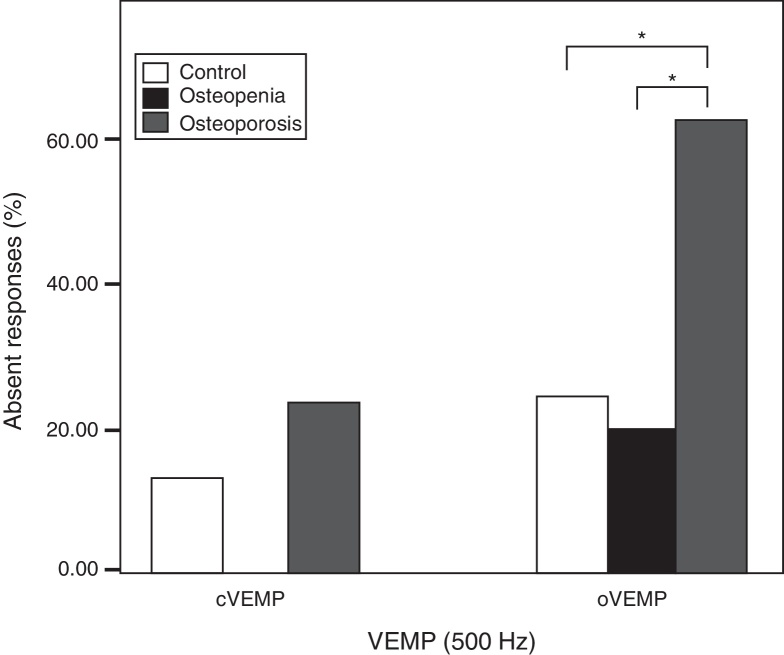
Response rates for cVEMP and oVEMP in the three groups. Stars indicate highly significant difference (*p* < 0.01).

The Equality of test for proportion was done to investigate the between the groups’ difference in response rate (number of ears with presence of cVEMP/oVEMP at 500 Hz) and frequency tuning. There was no significant difference in the response rate of cVEMP and proportions of frequency tuning at any of the frequencies (*p* > 0.1). However, significantly higher proportions of individuals with osteoporosis had complete absence of oVEMP than the control group (*Z* = 3.23, *p* = 0.001) and osteopenia group (*Z* = 3.54, *p* = 0.000). There was no significant difference in response rates of oVEMP between osteopenia and control group (*Z* = 0.36, *p* = 0.71). Fig. 4 shows the response rates of cVEMP and oVEMP across the groups and the outcome of the Equality of test for proportions.

### Outcomes of hearing assessment

All the participants underwent pure-tone audiometry, speech audiometry, immittance evaluation and Distortion Product Oto-Acoustic Emission (DPOAE). None of the participants had any conductive pathology, as this served as one of the exclusion criteria. There was a trend towards increase in Pure-Tone Average (PTA), SRT, resonance frequency and acoustic reflex threshold and reduction in SIS and DPOAE amplitude in the two clinical groups than the controls; however the groups were not significantly different on any of these tests (*p* > 0.1). The osteoporosis group revealed significantly higher proportions of ears with absent acoustic reflexes and distortion product oto-acoustic emissions than control group and osteopenia group (*p* < 0.05). There was a significant association between groups and SRT [*χ*^2^(2) = 35.7, *p* = 0.000], groups and SIS [*χ*^2^(2) = 131.85, *p* = 0.000] and groups and resonance frequency [*χ*^2^(2) = 46.49, *p* = 0.001] with more individuals with higher values of SRT and resonance frequency and lower value of SIS in two clinical groups than the control group.

### Association between hearing and balance

All the individuals in osteopenia and osteoporosis groups underwent audiological and balance evaluations. For studying the association between hearing and balance related deficits, the PTA of 20 dBHL or more was deemed an abnormal result (person had hearing loss) whereas the presence of abnormal findings on two of the three behavioural balance assessment tests (Fukuda stepping test, Tandem gait test and subjective visual vertical test) was considered an abnormal result. In terms of the objective vestibular assessment tests, absence of any one of cVEMP or oVEMP or both in one or both sides was considered an abnormal result. The Chi-square test revealed significant association between presence of hearing loss and abnormal result on behavioural balance assessment tests [*χ*^2^(1) = 4.82, *p* = 0.028] and marginally significant association between presence of hearing loss and abnormal result on objective vestibular assessment [*χ*^2^(1) = 3.45, *p* = 0.063].

## Discussion

In the present study significantly a higher proportion of individuals with osteoporosis reported vestibular symptoms than individuals with osteopenia or individuals in the control group. These findings are in agreement with those reported previously in this regard.^17^, ^19^ Older age has been shown to be associated with higher prevalence of vestibular symptoms.^18^, ^31^ However, there was no significant difference in age between the groups of the present study. Therefore, old age cannot explain these findings. These findings might be attributed to the reduction in calcium content within the vestibular system. The crista ampullaris present at the end of each of the semicircular canals and the maculae present in each of the otolith organs consist of large volumes of calcium (CaCO_3_).^20^ The studies on rats have shown the presence of ultra structural changes (decreased density) in the maculae of rats that had osteoporosis than in those which did not. Although there are no human studies reporting such ultra structural changes, similar changes might be associated with osteoporosis, which could in turn affect the maintenance of body balance.

### Comparison between the groups on behavioural tests

The results of SVV in the present study demonstrated a significantly higher proportion of abnormal results in the two clinical groups than the control group. SVV has been found to be a sensitive test for detection of utricularpathology.^32^, ^33^, ^34^ Therefore the results point towards the presence of utricular pathology in cases with osteopenia and osteoporosis. The utricular pathology in osteopenia and osteoporosis could be as a result of the reduction in calcium within the utricular macula. The utricular macula consists of large amounts of calcium carbonate crystals (otoconia) which form the major mass of the macula and have been reported to be vital for the activation (depolarization) or inhibition (hyper polarization).^35^ A study on lower animals with osteoporosis has shown ultra structural changes in the otoconia of the utricle.^20^ Although there are no studies of humans, a similar pattern of ultra structural changes in otoconia might be expected. Since the utricle plays a vital role in identifying the verticality of an object,^34^ the anatomical changes caused by osteoporosis and osteopenia in the utricle could have caused more deviation in the perceived vertical.

The results on other behavioural balance assessment tests (FST and TGT) revealed abnormal results in a significantly higher proportion of individuals with osteoporosis than osteopenia and the healthy controls. Although using other tests (CDP, Romberg and standing balance), studies showed higher sway amplitudes and more abnormal results in individuals with osteoporosis than healthy controls.^19^, ^31^ Thus this shows presence of vestibular pathology in individuals with osteoporosis. This again might be attributed to the ultra structural changes in the peripheral vestibular system^20^ as described above.

### Comparison between the groups on cVEMP and oVEMP

In the present study the results of objective assessment demonstrated a significantly higher proportion of abnormal results in individuals with osteoporosis than osteopenia and control group on oVEMP, but not on cVEMP. Although the process of ageing has been shown to be associated with reduction in amplitude and absence of Cvemp^36^ and oVEMP,^37^, ^38^ this probably could not have been the reason behind such finding in the present study as there was no significant age difference between the three groups of the present study. Further, the subject selection criteria ensured that subjects with conductive pathology were not included in the study. This was done because conductive pathology has been known to adversely affect VEMPs.^39^, ^40^ Therefore it also unlikely that absence of responses in more number of ears with osteoporosis was because of conductive hearing loss. Since oVEMP is mediated along the utriculo-occular pathway, and cVEMP along sacculo-collic pathway, the abnormal results on oVEMP further substantiate the presence of utricular pathology. It also points towards more robustness and lesser susceptibility of saccule and inferior vestibular nerve to the detrimental effects of pathology. The same has been confirmed through the findings of cVEMP and oVEMP in other vestibular pathologies like Benign Paroxysmal Positional Vertigo,^41^, ^42^ vestibular neuritis^43^ and auditory neuropathy spectrum disorders.^44^

An alternate thought worth discussing here is that of a possibility of the vestibular disturbances resulting in the whole process of bone remodelling. Some of the experimental evidences from lower animals like rats and mice tend to suggest that reduced vestibular functioning could actually be the cause of bone remodelling and thereby osteoporosis.^15^, ^16^ Whether this is true in human beings is a matter of debate and needs further experimental studies in humans with vestibular problems. Nonetheless, this could be an important result, if the evidence suggests.

### Association between hearing and balance deficits

The findings of the present study revealed significant association between the presence of hearing loss and presence of balance deficits. There are no studies, to the best of our knowledge, that have looked at such an association between hearing and balance in individuals with osteopenia and osteoporosis. This (significant association) indicates that people with reduced bone mineral density have hearing as well as balance related deficits and the two are closely related to each other. This further signifies the need for not only hearing evaluation but also for balance assessment in individuals with osteopenia and osteoporosis.

A close scrutiny of the overall results of balance testing shows a wide variety of results which suggests a spectrum rather than a single generalized condition. This would suggest a need for more comprehensive assessment and profiling of results on balance assessment in osteopenia and osteoporosis in order to develop a protocol in future.

Overall, the findings from the present study show that osteopenia and osteoporosis are associated with a higher prevalence of imbalance in general and vestibular dysfunction in particular. However, the study used only 35 participants split into three groups to arrive at such findings. This is not an appropriate sample size considering the prevalence of osteoporosis and osteopenia in the general population. Nonetheless, the study is a preliminary investigation and the findings are encouraging enough to warrant continued exploration in the area of balance and vestibular function testing with larger sample sizes.

## Conclusion

Findings of the present study are a testimony to the presence of balance-related deficits in individuals with osteopenia and osteoporosis. Therefore, clinical evaluations should include balance assessment as a mandatory aspect of the overall assessment so that a holistic management, which will be inclusive of the balance related rehabilitation, could be planned.

## Conflicts of interest

The authors declare no conflicts of interest.
